# Characteristics of Children With Persistent Pain and Their Parents in a Tertiary Interdisciplinary Pain Clinic

**DOI:** 10.1002/pne2.70005

**Published:** 2025-04-12

**Authors:** Ulla Caverius, Sophia Åkerblom, Jan Lexell, Marcelo Rivano Fischer

**Affiliations:** ^1^ Department of Health Sciences, Rehabilitation Medicine Lund University Sweden; ^2^ Department of Neurosurgery and Pain Rehabilitation Skane University Hospital Sweden; ^3^ Department of Rehabilitation Medicine Angelholm Hospital Sweden

**Keywords:** characteristics, functioning, parents, pediatric, persistent pain

## Abstract

Persistent Pain (PP) in children often has a high impact on their functioning. Knowledge about how to meet the needs is insufficient, especially regarding younger children, children with comorbid psychiatric health conditions, and within different national contexts. A specialized pediatric pain clinic for PP in Sweden offers assessment and interdisciplinary pain rehabilitation (IPR) and collects data on referred children and their parents in a registry. The aims of this study are to (i) describe clinically relevant aspects of children and parents at the first team assessment, (ii) investigate associations between symptoms of psychiatric comorbidity and functioning, (iii) investigate associations between symptoms of ADHD or symptoms of ASD and functioning, and (iv) describe the recommendations after the first team assessment and their rationale. A retrospective cohort study with a descriptive and exploratory design focusing on the characteristics of children (*n* = 510) and their parents at their first visit for specialized assessment at a tertiary pediatric pain clinic in Sweden between 2013 and 2021. Impairments and complexity appeared to increase with age, with high impact on daily and emotional functioning, especially in relation to symptoms of psychiatric comorbidity and ADHD or ASD. A majority of children and parents were uncertain about the cause of the pain. Only half of the children were recommended IPR, and numerous problems in functioning were found, not only related to pain. In agreement with previous studies describing characteristics of children with PP, there were more girls than boys and older than younger children at the first assessment. Both children and parents report several significant problems in physical, psychological, and social functioning, indicating a need for increased knowledge of PP and care in all kinds of pediatric health care and community settings. Tailored treatment interventions are recommended to improve functioning, including pain education, parental aspects, and addressing psychiatric comorbidities, with a special focus on ADHD or ASD symptoms. More thorough information to referring physicians about evaluations needed before referring to tertiary pain clinics could potentially help set the right expectations for further care and reduce the risk of diagnostic uncertainty.

## 1 Introduction

Persistent pain (PP) in children and adolescents (hereafter called children) is frequent, with a prevalence of 20%, and a large subgroup reports high impact on daily life and functioning [1, 2]. Over the years, pain medicine has moved from a biomedical view of pain to a multidimensional view, where pain is best described by a biopsychosocial model [3, 4]. In line with this, different domains to cover aspects of daily life and emotional functioning have been emphasized as core outcome domains in clinical trials on pediatric PP [5, 6]. Accordingly, a golden standard for the rehabilitation of complex persistent pediatric pain is a biopsychosocial approach with an individually tailored interdisciplinary pain rehabilitation (IPR) based on cognitive behavioral therapy (CBT) with the aim of increasing functioning and quality of life [2].

Highly impaired pediatric patients with pain in tertiary care have been described in the literature [7]. However, knowledge is still limited regarding characteristics of and risk factors for PP, particularly regarding younger children (< 10 years of age). Also for vulnerable children, such as those with comorbid psychiatric health conditions including attention deficit hyperactivity disorder (ADHD) and autism spectrum disorder (ASD), as well as children's caregivers (hereafter called parent) [2]. Published studies vary considerably in design and outcome measures. Most studies only describe the cohorts selected for IPR, leaving out the description of those not selected for IPR [8]. There is, to the best of our knowledge, a dearth of studies describing children referred to specialized tertiary care centers for assessment prior to selection regarding IPR, and the few published lack the parental aspect [7, 9]. Furthermore, it is challenging to generalize findings across studies and different national contexts [2, 5].

This study, based on data from a tertiary pain clinic in Southern Sweden offering biopsychosocial assessments and interdisciplinary rehabilitation (IPR) for children with PP, aspires to increase knowledge in this research area and remedy some of the above‐mentioned limitations. More specifically, the aims of this study are to (i) describe clinically relevant aspects of children and parents at the first team assessment, (ii) investigate associations between symptoms of psychiatric comorbidity and functioning, (iii) investigate associations between symptoms of ADHD or symptoms of ASD and functioning, and (iv) describe the recommendations after the first team assessment and their rationale.

## 2 Methods

### 2.1 Research Design

The present study is a retrospective cohort study with a descriptive and exploratory design focusing on characteristics of children with PP and their parents at their first visit for specialized assessment at a tertiary pain clinic located in the southern Sweden region. The current population of the region is about 2 100 000 people, representing about 20% of the total Swedish population.

Assessment tools in this study are selected according to suggested mandatory, important domains—pain severity/intensity, pain interference with daily life, overall well‐being, emotional functioning, physical functioning, and sleep quality, to cover biopsychosocial aspects of pain‐related impairments [5].

The study follows the Strengthening the Reporting of Observational studies in Epidemiology guidelines (STROBE) and contains the necessary items according to the STROBE checklist to properly report an observational study [10].

### 2.2 The Registry

The clinic keeps a registry that contains comprehensive information on physical, psychological, and social functioning, as well as quality of life, for children and their parents. This registry also includes sociodemographic data, details on symptoms of psychiatric comorbidities, pain characteristics, and information on analgesic use, derived from medical records spanning from the initial team appointment to the last follow‐up (for those attending IPR).

The self‐report questionnaires and assessment data on children and parents from their first team appointment is presented in Table 1. A yearly recruitment rate between 47 and 68 children resulted in 510 unique families between 2013 and 2021 who were included in the study.

**TABLE 1:** Reported variables to the registry.

Variables	Questionnaire	Child < 10 years	Child 10–18 years	Parent	By proxy parent all ages
Sociodemographics					
Sex	BGQ	X	X	X	
Age	BGQ	X	X	X	
Marital status	BGQ			X	
Highest education	BGQ			X	
Employment status	BGQ			X	
Pain characteristics					
Pain intensity	NRS	X	X		
Pain locations	Pain chart	X	X		
Pain explanation received	BGQ		X	X	
Physical functioning					
Functional disability	FDI		X		X
Physical functioning	PedsQL_phys_.		X		X
Psychological functioning					
Depression	CES‐DC		X		
Anxiety	SCARED‐R		X		
Insomnia	ISI		X		X
Pain‐related worrying	PRS		X	X	
Psychological inflexibility	PIPS		X		
Psychological flexibility	PPFQ‐10			X	
Social functioning					
Pain interference	PII		X		X
School absence	BGQ				X
Quality of life					
Quality of life	PedsQL_tss_		X		X

Abbreviations: BGQ, Background Questionnaire; CES‐DC, Center for Epidemiological Studies—Depression Child; FDI, Functional Disability Inventory; ISI, Insomnia Severity Index; NRS, Numeric Rating Scale; PedsQL_phys_, Pediatric Quality of Life Inventory physical functioning; PedsQL_tss_, Pediatric Quality of Life Inventory total scale score; PII, Pain Interference Index; PIPS, Psychological Inflexibility in Pain Scale; PPFQ‐10, Parental Psychological Flexibility Questionnaire revised and shortened version; PRS, Pain Reactivity Scale; SCARED, Screen for Child Anxiety Related Emotional Disorder—Revised.

### 2.3 Procedures

At the first assessment, children and parents are interviewed and assessed separately by a pain physician, a physiotherapist, and a psychologist. Both the child and their parents are assessed together as well as separately by the psychologist. A team discussion leading to a decision about recommendations follows the assessments. The family then receives a summary and a dialogue about the team discussions and recommendations, including a brief explanation of the pain condition and its complexity.

The children and their parents are instructed to fill in requested questionnaires at home and bring them to the first assessment. Additional data are obtained from the medical records.

### 2.4 Participants

Children were usually referred by pediatricians, general physicians, or specialists in pediatric orthopedics, neurology, gastroenterology, and psychiatry. All children underwent medical evaluations by the referring physician or other previous care providers. Interpreters were offered to all non‐Swedish speaking families or families not fluent in Swedish. Children were included in the registry if (1) the family approved the data being saved in the registry, (2) the child had suffered from pain continuously or at least once a week for > 3 months, (3) they were referred to the Specialized Pediatric Pain Clinic (SPPC), and (4) the referral team at the SPPC had approved the referral containing medical history, information that the investigation had been completed, and the referrer's assessment. Incomplete referrals were returned or completed with a call to that referrer if (1) assessment or diagnosis was missing, (2) other investigations or treatments were ongoing or planned elsewhere, if the treatment was suspected of interfering with an IPR, or (3) impairments of daily life and functioning were explained by another condition such as psychiatric comorbidity or somatic disease.

Parents were invited to participate in the first visit, and all children had at least one parent participating and answering the routine set of questionnaires.

With no exception, all families (*n* = 510) accepted to be included in the registry.

### 2.5 Child Measures

The study adapted the recommended outcome domains for pediatric PP research as far as possible [5]. For children older than 10 years of age, self‐report questionnaires were used for evaluation, and for children younger than 10, parental by‐proxy reports of their child were used.

#### 2.5.1 Demographics

For all participants (children and parents), their sex and age were registered. There were no children younger than 6 years referred for assessment. Since there is a shortage of studies on younger children, children < 10 years of age were included in the study and analyzed with questionnaires by proxy parents. In comparisons between children of younger and older ages, the latter was defined as children between 10 and 18 years.

#### 2.5.2 Pain Characteristics

All participants were considered to have PP, defined as continuous pain or pain recurring more than once a week for at least 3 months. The following pain characteristics were included: pain type, pain location, pain duration from onset, pain intensity, pain medication at regular times and as needed (use of opioids was noted) and diagnostic uncertainty.

***Pain types*** have in the registry been divided into nociceptive, neuropathic, nociplastic, or idiopathic pain [11, 12].

***Pain Location*** A pain chart with 21 body locations was used to obtain self‐reported pain locations. The participants were asked to mark all areas of the body with pain and to mark the main location/s with an “X” [13], which was reported as the primary pain location/s in the registry.

***Pain Intensity*** *Numerical Rating Scale* (NRS) was used to assess pain intensity. NRS is graded 0 to 10, with 0 representing “no pain” and 10 “worst pain imaginable” Current, average last week, highest and lowest pain were self‐reported [14, 15].

***Pain Medication*** In the background forms, the parents reported which current medications for pain the child was treated with, both regularly and as needed. Both the type and number of different medicines were registered in the registry.

***Diagnostic Uncertainty*** Uncertainty about the cause of pain was investigated by asking in the background forms whether the child received any explanation for his/her pain, yes or no. Both children and parents answered the question separately.

#### 2.5.3 Symptoms of Psychiatric Comorbidities

Mental well‐being was registered in terms of symptoms of possible psychiatric comorbidities (i.e., depression, anxiety, insomnia, ADHD, or ASD). Self‐report questionnaires were used to assess depression, anxiety, and sleep difficulties. Values above the cut‐off in questionnaires for depression, anxiety, and sleep difficulties were considered to signal possible psychiatric comorbidity. As several of these forms have not been validated for younger children, only children ≥ 10 years of age have been included in the analyses of symptoms of psychiatric comorbidity.

Symptoms of ADHD or ASD were defined as having a diagnosis or being under investigation for ADHD or ASD. Information about ADHD or ASD was obtained and registered at the first assessment visit.

#### 2.5.4 Self‐Report Measures (Covering Physical, Psychological, and Social Functioning)

*Functional Disability Inventory* (FDI) evaluates the impact of illness on children's physical and psychosocial functioning [16]. The FDI was used to assess physical functioning, as was earlier recommended by PedIMMPACT [17] for assessing physical functioning from school age. Current recommendations for measuring physical function are not available in Swedish yet [6]. FDI consists of 15 questions rated 0–4, where total cut‐off scores are set to no/minimal (0–12), moderate [13, 14, 15, 16, 17, 18, 19, 20, 21, 22, 23, 24, 25, 26, 27, 28, 29], or severe disability (≥ 30). The maximum score is 60 [18]. Excellent reliability and validity are reported regarding FDI in pediatric studies of PP [19], and a Swedish study on the validity and reliability of the questionnaire for children 8–17 years old found high internal consistency reliability, with Cronbach's *α* 0.90 [20].

*Center for Epidemiological Studies—Depression Child* (CES‐DC) assesses depressive symptoms in children. CES‐DC is a 20‐item scale with a maximum score of 60; greater scores indicate greater depressive symptoms. The scale is validated internationally for children to young adults, 6–23 years old, with a Cronbach's reliability coefficient *α* of 0.76 for younger children and 0.86 for older children and young adults [21]. Validation of CES‐DC in a Swedish study of adolescents indicated that using a cut‐off score of 24 and above, the CES‐DC was specific enough for detecting depressive disorder. In the Swedish study Cronbach's reliability coefficient *α* was 0.91 (0.91 for girls and 0.87 for boys) [22].

*Screen for Child Anxiety Related Emotional Disorder—Revised* (SCARED‐R) assesses anxiety symptoms. The SCARED‐R is a 50‐item questionnaire, each item scoring 0 to 2 (0 = almost never, 1 = sometimes and 2 = often), with a maximum score of 100. The SCARED‐R is a valid and reliable screening tool for any anxiety disorder or specific anxiety disorder, with a cut‐off score of 25 for any anxiety disorder. It is used internationally and nationally for clinical and research purposes [23].

*Insomnia Severity Index* (ISI) assesses insomnia. The ISI consists of seven items concerning sleep problems, rated from 0 (not a problem/not at all) to 4 (very much), with a maximum score of 28. Higher scores indicate more problems, with a cut‐off score of 9 for clinically significant insomnia [24]. A Swedish study used the cut‐off of 9 for validation of ISI for children with PP 10–18 years old and found an internal reliability, Cronbach's *α* = 0.88 [25]. ISI is a reliable and valid instrument, used also in several international studies for both adults and children [24, 26].

*Pain Interference Index* (PII) was used to assess social functioning. PII assesses the impact of pain on daily activity and physical, psychological, and social functioning. It is a six‐item questionnaire, addressing six areas of daily life: school, friends, curricular activities, mood, physical activities, and sleep, and to what extent pain interfered with these areas over the last 2 weeks. Each item is rated from 0 (not at all) to 6 (completely), with a maximum score of 36. The scale has shown good psychometric properties for adults as well as children with PP, being used in national and international studies with symptoms of good sensitivity to change. Internal consistency of items in PII measured by Cronbach's *α* was 0.86 in a Swedish study of children with PP aged 7–18 years [27], and the scale has been used in national and international studies for both children and adults with symptoms of good sensitivity to change [27, 28, 29].

*Psychological Inflexibility in Pain Scale* (PIPS) assesses psychological inflexibility related to pain. Psychological flexibility is defined as the ability to contact the present moment more fully, and to change or persist in behavior when doing so serves valued ends [30]. PIPS consists of 12 items with two subscales: avoidance (eight items) and fusion (four items) rated from 1 (never true) to 7 (always true). The maximum total score is 84; greater scores indicate higher levels of psychological inflexibility. The PIPS is validated for children in an Iranian study with a Cronbach *α* of 0.66 [31]. PIPS has shown satisfactory psychometric properties in studies on adults in Sweden and may be used for exploring psychological inflexibility in PP and to analyze processes of change, and for tailoring interventions [32, 33]. Although not validated in Swedish for children, the form is clinically used for children in Sweden.

*Pain Reactivity Scale* (PRS) assesses the degree of psychological reactivity, i.e., reacting with worry and general emotional reactivity to pain. PRS consists of five questions to be answered according to a scale from 0 (not at all) to 6 (always), with a maximum score of 30; greater scores indicate more discomfort. PRS has satisfactory psychometric properties and is being used in several Swedish studies on pediatric PP and functioning. There are indications that pain‐related worrying is important for explaining variance in pain interference, and PRS shows sensitivity to change and affects functional outcomes after ACT treatment [29, 34].

The *Pediatric Quality of Life Inventory* (PedsQL 4.0 Generic Core Scales) assesses overall well‐being. PedsQL encompasses four subscales: physical functioning (eight items), emotional functioning (five items), social functioning (five items) and school functioning (five items). Items are reverse‐scored and linearly transformed to a 0–100 scale. Greater scores indicate better health‐related quality of life (HRQoL). A cut‐off score of 83 was used to identify children younger than 8 years old with special health care needs or chronic conditions. Cut‐off scores for moderate chronic conditions were 79 and 77 for major chronic conditions. For children 8 years and older, the cut‐off scores were 78, 76, and 70, respectively [35]. PedsQL self‐report has shown high validity and reliability in international studies as well as a Swedish study on school children, with internal consistency reliability, Cronbach's *α* of > 0.7 for all subscales and close to 0.9 for the total score scale in both [36, 37].

### 2.6 Parent Measures

Besides information on parents' sociodemographic situation and report of them having or not having PP, the registry contains information from two categories of parental questionnaires: self‐report and child by‐proxy report. For the study, child by‐proxy reports were only used for description of children under the age of 10.

#### 2.6.1 Sociodemographics and Parental Pain

Sociodemographic data on parents (sex, age, marital status, occupational status), information about their own PP as well as their pain‐related distress were obtained from the quality registry.

#### 2.6.2 Self‐Report Measures

*Pain Reactivity Scale* (PRS‐p) is a self‐report questionnaire rating the parent's own reaction to the child's pain. The same items as for children are rated, but from the perspective of the parent; for example, “How often do you worry about your pain” is transformed to “How often do you worry about your child's pain”. For grading, see PRS child above [29, 38].

*Parental Psychological Flexibility Questionnaire* (PPFQ‐10) assesses parent's response to adolescent PP that includes the process of psychological flexibility, a main target of ACT‐interventions. Psychological flexibility is defined as the parent's willingness to experience distress related to the child's pain in the service of valued behavior. The PPFQ as well as the revised and shortened version PPFQ‐10, translated to Swedish, are valid and reliable instruments, and used in national and international studies. PPFQ‐10 has shown good internal consistency reliability [39, 40, 41], Cronbach's *α* = 0.86, in a Swedish study of children with PP [41]. PPFQ‐10 items are rated from 0 (never true) to 6 (always true) with a maximum score of 60.

#### 2.6.3 Child by Proxy Parent

For children under the age of 10, reports by proxy parents were used, since not all questionnaires have been checked for validity and reliability for younger children.

*School absence* related to pain was reported by parents, either as days per month or percentage per semester. Absence was noted in the registry as percentage and divided into groups: 0%, 1%–20%, 21%–99%, or total school absence.

*Functional Disability Inventory* (FDI‐p) is a proxy report rating the child's physical and psychosocial disability during the last 2 weeks. The scale has shown satisfactory validity and reliability in international studies, and good agreement between ratings from children and parents. An American study evaluating psychometric properties of FDI in children with PP found the internal consistency reliability, Cronbach's *α*, for parent‐report FDI for parents of girls (*α* = 0.94) and boys (*α* = 0.90) [19]. For scoring, see FDI child version above; greater scores indicate greater disability.

*Pediatric Quality of Life Inventory* (PedsQL 4.0 Generic Core Scales) (by proxy) assesses parents' perceptions of their child's HRQoL. PedsQL parent‐proxy report demonstrates reliability and validity at the individual age group 2–16 years. Since there is low agreement between child and parent ratings in both national and international studies, by proxy report PedsQL is only recommended to complement the child self‐report as a secondary outcome measure, not as a substitute or proxy for pediatric self‐reports [36, 42]. For very young children or those unable to self‐report, a parent‐proxy report can be used as a primary outcome measure. For scoring, see child PedsQL self‐report [37, 43].

*Pain Interference Index* (PII‐p) is a parent‐proxy report rating to what extent pain interferes with the child's daily life. PII and PII‐p have shown to be reliable, valid, and feasible measures to assess pain interference in children and adolescents ages 6–25 years with chronic illness. Internal consistency reliability for the English version of PII children was 0.84 and 0.96 for PII parents [44]. For scoring, see PII self‐report.

### 2.7 Recommendations

The recommendations for further treatment after the first team assessment were categorized as follows: 1. IPR at the clinic, 2. Advice to the referring clinic, 3. Referral to child psychiatry for assessment, 4. Offered IPR, but the family refused the offer, or 5. Other, e.g., short‐term unimodal contact with a physiotherapist at SPPC or referral to adult pain rehabilitation.

### 2.8 Ethical Considerations

All families were informed about the registry and gave, without exception, opt‐out consent to allow for the storage of information and for data to be used in future research, pending approval from the Ethical Research Board. Families were also informed that denying consent would not in any way influence the recommendations or the treatments offered at the clinic. We have carefully considered the vulnerability of children taking part in research and the fact that it is the parents who gave informed consent. The study was approved by the Swedish Ethical Review Authority 2021‐07‐26 (No. 2021–02709). Throughout the study, the Declaration of Helsinki for research on humans was followed.

### 2.9 Data Quality Control and Statistical Analyses

Ten percent of all registrations were randomly checked and compared with the questionnaires and medical records, with an error rate of less than 0.003%, indicating that the quality control was satisfactory.

In the present study, sociodemographic, child, and parent characteristics were presented by means of frequency statistics for categorical variables and standard deviation statistics for continuous variables. Descriptive data for the children were presented by age groups (< 10 years; 10–12 years; 13–15 years; 16–18 years). Children < 10 years are referred to as “younger” and those 10–18 years are referred to as “older.” Missing data were excluded from the analyses because fewer than 3% missing data was found for all variables, except anxiety and ADHD/ASD, for which a larger amount was missing completely at random due to administrative problems. Valid percentage was used for calculations.

Two analyses were performed to examine possible relationships between mental well‐being and functional level in older children. In the first, it was investigated whether the level of functioning was related to symptoms of high or low psychiatric comorbidity. Signs of high comorbidity were defined as two or more answered questionnaires (CES‐DC, SCARED‐R or ISI) above cut‐off or presence of symptoms of ADHD or ASD and at least one or more answered questionnaires above cut‐off. Signs of low comorbidity were defined as less than two psychiatric comorbidities (CES‐DC, SCARED‐R, ISI, symptoms of ADHD, or symptoms of ADS). Secondly, a separate analysis was conducted to investigate whether symptoms of ADHD or ASD affected levels of functioning, depression, anxiety, and insomnia. For both analyses, independent t‐tests were used for comparison of parametric variables and the Mann–Whitney *U*‐test for non‐parametric variables. The Bonferroni corrections were applied throughout these analyses.

Throughout, *p*‐values less than 0.05 were considered statistically significant. All statistical analyses were performed with IBM SPSS Statistics Software v. 29 (IBM Corporation, Amonk, NY, USA).

## 3 Results

### 3.1 Description of Children and Parents

Descriptive statistics were used to investigate the characteristics of children and parents.

Child variables reported and analyzed are summarized in Table 2. More than twice as many girls as boys consulted the SPPC. Only a few children were under the age of 10, and none under 6 years of age.

**TABLE 2:** Characteristics of children consulting a specialized pediatric pain clinic.

Variables		Ref. interval	< 10 yrs., *N* = 26	10–12 yrs., *N* = 114	13–15 yrs., *N* = 195	16–18 yrs., *N* = 175
Demographics						
Sex—female	*N* (%)		18 (69)	71 (62)	134 (69)	144 (82)
Pain characteristics						
Primary pain location	*N* (%)					
Headache/orofacial			2 (8)	36 (32)	56 (29)	48 (27)
Abdominal			11 (42)	37 (33)	41 (21)	19 (11)
Wide‐spread			0	9 (8)	22 (11)	41 (23)
Other (e.g., extremities)			7 (27)	21 (18)	37 (19)	27 (15)
Back and/or neck			2 (8)	5 (4)	25 (13)	27 (15)
Joint			4 (15)	6 (5)	14 (7)	13 (7)
Number of pain locations	*N* (%)					
One			16 (62)	54 (47)	66 (34)	42 (24)
Three or more			7 (27)	46 (40)	97 (50)	119 (68)
Pain type—dominating nociplastic or idiopathic	*N* (%)		25 (96)	112 (98)	184 (94)	164 (95)
High pain intensity (NRS) ≥ 5	*N* (%)	0–10	8 (31)^a^	66 (58)	113 (58)	120 (88)
Pain explanation: yes	*N* (%)		9 (43)^b^	59 (52)	78 (39)	67 (39)
Duration in months since pain started	Mean (SD)		42 (31)	40 (33)	46 (37)	58 (44)
Pain medication	*N* (%)					
Daily regularly			3 (11)	5 (3)	5 (3)	5 (2)
As needed			9 (35)	60 (53)	119 (62)	107 (62)
Opioids^c^ regular			0	0	0	1 (1)
Opioid^c^ as needed			1 (4)	2 (2)	5 (3)	14 (8)
Physical functioning						
Functional disability (FDI)	Mean (SD)	0–60	11 (8)^b^	17 (12)	17 (10)	20 (12)
FDI no/mild	*N* (%)	0–12	16 (64)^b^	46 (40)	72 (37)	59 (34)
FDI moderate	*N* (%)	13–29	7 (28)^b^	49 (43)	98 (51)	83 (48)
FDI severe	*N* (%)	30–60	2 (8)^b^	19 (17)	24 (12)	30 (17)
Physical functioning (PedsQL_phys_)	Mean (SD)	0–100	56 (24)^b^	50 (22)	49 (20)	47 (19)
Psychological functioning						
Depression (CES‐DC)^d^	*N* (%)	≥ 24	n.a.	22 (29)	34 (28)	46 (42)
Anxiety (SCARED‐R)^d^	*N* (%)	≥ 25	n.a.	n.a.^f^	37 (37)^f^	51 (53)^f^
ADHD or ASD^d^, ^e^	*N* (%)		n.a.	11 (14)	25 (20)	21 (19)
Insomnia (ISI)^c^	*N* (%)	≥ 9	n.a.	28 (36)	52 (42)	65 (59)
Psychiatric comorbidity ≥ 2^d^	*N* (%)		n.a.	25 (33)	58 (47)	61 (56)
Psychological inflexibility (PIPS)	Mean (SD)	12–84	n.a.	49 (14)	50 (15)	54 (15)
Pain reactivity/worrying (PRS)	Mean (SD)	0–30	n.a.	14 (7)	16 (8)	18 (8)
Social functioning						
Pain interference (PII)	Mean (SD)	0–30	14 (19)^b^	15 (9)	16 (9)	19 (8)
School absence %, total	Mean (SD)		9 (15)	27 (30)	33 (34)	30 (32)
School absence > 1 d/w	*N* (%)		5 (19)	52 (46)	94 (49)	84 (50)
Quality of life						
Quality of life (PedsQLtss)	Mean (SD)	0–100	64 (18)^b^	59 (16)	55 (16)	53 (16)

*Note:* Self‐report and assessment data by age groups at the first visit (*n* = 510).

Abbreviations: CES‐DC, Center for Epidemiological Studies–Depression Child; FDI, Functional Disability Inventory; ISI, Insomnia Severity Index; NRS, Numeric Rating Scale; PedsQL, Pediatric Quality of Life Inventor; PII, Pain Interference Index; PIPS, Psychological Inflexibility in Pain Scale; SCARED‐*R*, Screen for Child Anxiety Related Emotional Disorder–Revised.

^a^ NRS average last week, except for children < 10 years where the current NRS at the first visit was noted.

^b^ Children by‐proxy parent data.

^c^ Opioids = opioid, codeine, tramadol.

^d^ No data on anxiety and ADHD OR ASD before 2016, why all psychiatric comorbidities are calculated between 2016 and 2021 and *n* = 314.

^e^ ADHD or ASD, diagnosed or under investigation for.

^f^ Missing data due to random logistics failures.

High pain intensity was frequently reported by older children, progressively increasing with age. Furthermore, 88% of the children in the age group from 16 to 18 years reported high pain intensity. Mean pain duration for all children was 4 years. The main pain types were idiopathic or nociplastic pain. The most prevalent primary pain location was headache for the older children and abdominal pain for the younger children. Most older children reported three or more pain locations, while a single pain location was often reported by younger children. More than half of the older children had not received an explanation for their pain. Few children reported regular intake of analgesics, with medication as needed being more usually reported. Almost no opioids were used.

The mean FDI score for assessment of physical functioning in children aged 10 to 18 was 18 (SD ±11) and 303 (63%) reported at least moderate impact. Younger children reported lower values for mean FDI, 11 (SD ±8), and the majority were categorized into no/mild severity, indicating that physical functioning is deteriorating with age. The mean percentage of physical functioning, assessed with the subscale PedsQL physical functioning, was 56% for younger children and 49% for older children.

School absenteeism was generally high in older children, with more than 1 day per week absence reported by nearly 50% and total absence by 9%. High levels of pain interference (PII) were reported by all age groups. A mean of 51 (SD ±15) was reported by older children in psychological inflexibility total score (PIPS) and a mean of 16 (SD ±8) for pain‐related worrying (PRS).

Symptoms of psychiatric comorbidity were common in older children with depression in 33%, anxiety in 44%, and clinically significant insomnia in 47% of all children 10–18 years old. The age group 16–18 years stood out with the highest percentages—depression 42%, anxiety 53%, and insomnia 59%—with increasing cases throughout the age groups. As many as 56% in this age group were found to have symptoms of ≥ 2 psychiatric comorbidities, compared to 33% and 47% in age groups 10–12 and 13–15, respectively.

Table 3 summarizes data on the characteristics of the parents of children with PP before the first appointment. Of the parents answering the questionnaires, 81% were mothers, and most parents accompanying their child to the clinic were also mothers, of which more than half were well‐educated and working. Less than 20% of the reporting parents reported some level of sick leave or disability pension. More than half of all mothers and a third of all fathers suffered from PP. The majority expressed uncertainty about the cause of their child's pain. Parents scored high for pain‐related worrying for their child's pain and low for psychological flexibility, indicating dysfunctional coping strategies.

**TABLE 3:** Demographic and pain characteristics of parents of children with persistent pain before the first appointment at a tertiary pediatric pain clinic in Sweden.

Variables		*N* (%)
Sociodemographics		
Sex of reporting parent—female		407 (81)
Age	Mean (SD)	45.4 (6)
Highest education		
High school or less		223 (45)
College or university		277 (55)
Marital status		
Married or registered partnership		333 (66)
Partner		77 (15)
Live‐apart relationship		22 (4)
Single or widow or widower		71 (14)
Employment status		
Full‐time work or study		319 (63)
Part‐time job or study		92 (18)
Reporting sick or disability pension full or part time		83 (17)
Other (jobless, parental leave, no wish to work)		9 (2)
Parental pain characteristics		
Persistent pain	Mother	277 (56)
	Father	170 (37)
With no impact on working ability	Mother	184 (66)
	Father	147 (86)
With impact working ability	Mother	93 (34)
	Father	23 (14)
Pain explanation regarding child: yes		212 (42)
Parental distress		
Worry for the child's pain (PRS) 0–36	Mean (SD)	18.8 (7.4)
Psychological flexibility (PPFQ) 0–60	Mean (SD)	28.9 (11.4)

*Note:* *N* = 504.

### 3.2 Associations Between Symptoms of Psychiatric Comorbidity and Functioning

There was a varying degree of impairment related to levels of psychiatric comorbidity in children aged 10–18 years old, as shown in Table 4. *T*‐tests and Mann–Whitney U tests were conducted to examine differences in functioning between children with high or low symptoms of psychiatric comorbidities. Children with symptoms of high psychiatric comorbidity (47%) had higher pain intensity, pain interference, and psychological inflexibility, lower quality of life, and more functional disability than children with symptoms of low comorbidity (53%). These differences were significant after Bonferroni correction was performed (adjusted *α* = 0.008). No significant difference in school absence was revealed between children with high and low psychiatric comorbidity.

Differences in impairments between children with symptoms of and children without symptoms of ADHD or ASD, age 10–18 years, are presented in Table 5. *T*‐tests and Mann–Whitney U tests were conducted to examine differences in functioning between children with or without symptoms of ADHD or ASD. About 20% reported symptoms of ADHD or ASD, and these children had significantly higher pain interference and school absence as well as significantly more symptoms of depression, insomnia, functional disability, and lower quality of life than children without symptoms of ADHD or ASD.

**TABLE 4:** Impairments by symptoms of high and low psychiatric comorbidities in children 10–18 years of age between 2016 and 2021.

Variable	M (SD)/median (q_1_, q_3_)^a^		Mean diff.	*p*
	Group 1 low, *N* = 163	Group 2 high, *N* = 144		
Pain intensity (last week)	5.97 (1.97)	6.88 (1.89)	−0.91	< 0.001^b^
Pain interference	12.59 (7.57)	21.15 (7.86)	−8.57	< 0.001^b^
Psychological inflexibility	45.91 (14.18)	57.22 (13.64)	−11.31	< 0.001^b^
Quality of life	64.17 (13.63)	45.93 (13.10)	18.23	< 0.001^b^
Functional disability	13.80 (9.55)	22.99 (10.37)	−9.19	< 0.001^b^
School absence %	18.00 (0.00, 45.50)	25.00 (4.75, 67.75)		0.012^c^

*Note:* *α* = 0.05. Group 1 (low) = less than two psychiatric comorbidities. Group 2 (high) = two or more psychiatric comorbidities.

^a^ Independent *T*‐test was performed for all variables of impairments except for school absence, where the Mann–Whitney *U*‐test was used to evaluate impairments regarding high or low psychiatric comorbidity.

^b^ Significant even when the Bonferroni correction is applied.

^c^ Non‐significant when the Bonferroni correction is applied.

**TABLE 5:** Impairments by the presence or absence of symptoms of ADHD or ASD in children 10–18 years of age between 2016 and 2021.

Variable	M (SD)/median (q_1_, q_3_)^a^		Mean diff.	*p*
	Group 1—no, *n* = 256	Group 2—yes, *n* = 57		
Pain intensity average	6.35 (2.03)	6.62 (1.70)	−0.27	0.367
Pain interference	15.89 (8.77)	19.84 (8.31)	−3.95	0.002^b^
Psychological inflexibility	50.53 (15.21)	54.02 (13.86)	−3.49	0.118
Quality of life	57.40 (15.85)	47.77 (15.27)	9.64	< 0.001^b^
Functional disability	17.20 (10.67)	22.29 (11.18)	−5.09	0.002^b^
Depression	22.59 (12.30)	29.13 (11.46)	−6.54	< 0.001^b^
Anxiety	23.04 (16.67)	29.88 (18.39)	−6.84	0.022^c^
Insomnia	8.45 (6.85)	11.41 (7.17)	−2.96	0.004^b^
School absence %	15.00 (0.00, 50.00)	50.00 (20.00, 100.00)		< 0.001^b^

*Note:* *α* = 0.05.

^a^ Independent *T*‐test was performed for all variables except for school absence, where the Mann–Whitney *U*‐test was used to evaluate impairments by the presence of symptoms of a neuropsychiatric condition.

^b^ Significant even when the Bonferroni correction is applied.

^c^ Non‐significant when the Bonferroni correction is applied.

### 3.3 Recommendations

The purpose of the first assessment at the clinic was to identify the recommendations that the child would benefit from. Half of the children after the first assessment were offered and accepted the recommendation of IPR at the clinic. Of the other half, 25% were returned to the referring clinic with advice. The reasons were: (a) spontaneous improvement in functioning, (b) pain recovery, (c) family requests for other treatments than IPR, such as further assessments, individual physiotherapy, or medication, or (d) identification of a need for interventions targeting family functioning. The team referred 15% to pediatric psychiatry for assessment on suicidality, explicit anxiety, or self‐harming behavior. Very few declined the offer of IPR (7%).

## 4 Discussion

Summarizing the main results, we found impairments and complexity increasing with age, and high impact on daily and emotional functioning, especially in relation to symptoms of psychiatric comorbidity and symptoms of ADHD or ASD. The majority of children and parents were uncertain about the cause of the pain. Only half of the children were recommended IPR, and numerous problems in physical, psychological, and social functioning were found, not only related to pain. In agreement with previous studies describing characteristics of children with PP, there were more girls than boys and older than younger children at first assessment at the clinic. Although the increased impairment and complexity in older children have been described earlier [7], this study confirms previous results and clearly shows how the complexity gradually increases with age.

Headache was the main pain location for older children and abdominal pain for younger ones, in agreement with previously published prevalence studies [7, 45]. Multiple pain locations, generalized pain, and high pain intensity were more frequent in older children than in younger ones, gradually increasing with age, in accordance with results from the limited number of studies focusing on first assessment in tertiary care [7, 9].

To the best of our knowledge, this is the first study reporting age‐related school absence in children referred to tertiary care, where only 19% of younger children reported high school absence but nearly 50% of older children. Results from previous studies of children aged 6–18 found high school absence in 25%–30% for the whole group, independent of age [7, 9]. Taken together, our results could reflect a process of deterioration as pain spreads, the number of locations and the intensity increase while school participation and levels of functioning decrease, emphasizing the need for early prevention and intervention regarding pain. School is one of the natural social platforms for children, and high school absence can be an important factor eventually leading to exclusion from further education, work possibilities, and decreased self‐esteem. Consequently, school interventions should be a mandatory part of the IPR when school absence exists.

We found no evidence of long‐term opioid and analgesic overuse in contrast to studies from other national contexts [7, 46], indicating that Swedish pediatricians are reluctant to prescribe opioids. Although children often have a history of many medications prescribed, very few seem to continue ineffective drugs on a regular basis.

Psychiatric comorbidity in pediatric PP remains understudied. In line with our results, the few published studies focusing on psychiatric comorbidities in similar populations reported greater pain interference for children with depression [9], and lower resilience in children with various psychiatric comorbidities compared to children without comorbidities [47]. The findings in this study expand on the literature by suggesting that symptoms of psychiatric comorbidities are common in children with PP in tertiary care and that there is an association between symptoms of psychiatric comorbidity and poorer levels of functioning. We found that children with high psychiatric comorbidity reported greater impact on all measures of physical, psychological, and social functioning than children with no or low psychiatric comorbidity, emphasizing the importance of including psychiatric symptoms as influential factors in the biopsychosocial model of chronic pain. Our results also suggest that screening for psychiatric comorbidities should be included in standard assessment of PP in children and be targeted when tailoring treatment for children with PP. The mean score for school absence in the presence of symptoms of ADHD or ASD was 35% greater than in the absence of symptoms of ADHD or ASD, which strongly implies that collaboration with the school is a key component of IPR for children with ADHD or ASD symptoms. This pediatric patient group (ADHD or ASD and PP) is typically seen in both somatic and psychiatric settings [48, 49] emphasizing the need for knowledge of biopsychosocial perspective, including pain management, in all pediatric care. The existing literature on children with ADHD or ASD is scarce [50]. Thus, the tertiary pain clinic at Skåne University Hospital could be considered an exception, having proactively integrated screening for ADHD or ASD into our assessment and may serve as an inspiration for other tertiary pain settings.

Many children and parents reported uncertainty about the cause or explanation for the pain, defined as diagnostic uncertainty (DU) [51, 52]. Our study, in agreement with studies in North America [53, 54], is the first to report the prevalence of DU in children with PP in a European context. DU is an increasing area of interest in pediatric studies. Adult studies have shown relations between DU and pain catastrophizing, emotional distress as well as increased pain‐related disability [55] and similar associations have been hypothesized for children and their parents [53, 54]. It can be argued that education on pain for both providers and families might be an important intervention to counteract DU. It seems that outside clinics are sometimes not adequately preparing families for pain clinic referral, e.g., due to ongoing medical evaluations, lack of feedback on performed evaluations and results, and insufficient information about the shift to pain rehabilitation, in favor of increased functioning rather than pain relief. To decrease the risk of diagnostic uncertainty and increase motivation for IPR, we believe it is crucial that the evaluation of any underlying causes of the pain is completed prior to referral. The results of the assessments should be clearly communicated to the family, along with information that further diagnostic investigations are not indicated before referral. At the same time, the child's suffering must be validated. Information to referring physicians about the importance of providing sufficient information to the families needs to improve, including regular updates. In Sweden, since 2023 there is a national care program for children and young people with chronic pain, describing what is expected at different levels of care regarding investigation, recommended treatment, information to the family, and referral to different levels of care [56].

Reported quality of life for both younger and older children in this study was lower than both a control group of healthy children [57] and a study with school children [58]. These findings highlight the complexity of the problems faced by the children and the need for access to IPR to stop a negative downward spiral in their functioning and quality of life.

In contrast to some studies [59], yet confirming others [38, 60], parents reported high educational levels and employment rates, potentially suggesting a selection bias for referral to pain rehabilitation due to socioeconomic status.

Many parents in our study suffered from PP themselves, mothers more often than fathers. It has been noted that PP in parents can influence the onset and persistence of PP in their children [61]. Decrease in parental distress for both mothers and fathers after intensive IPR including parental interventions has been reported [62]. This indicates the importance of engaging parents in their child's pain rehabilitation that also include interventions targeting parents' own well‐being and well‐functioning parenting [63, 64].

Affected psychological flexibility, pain‐related worry, and DU were found among the parents. Parenting a child with PP can be distressing and linked to more protective behavioral patterns than wished for, threatening the normal course of developing independence of the child. Parental distress and protective behavior have been shown to affect child functioning in previous studies [65, 66].

Mostly mothers participated in visits to the SPPC, leaving fathers' characteristics relatively unexplored, paralleling previous studies [38, 67]. Nevertheless, the frequency (19%) of participating fathers was fairly high compared to earlier studies that, with few exceptions, failed to include information on paternal‐related outcomes [68, 69]. Sociocultural differences might partly explain the higher frequency of fathers involved in this study as compared to studies from other countries, since the family policy in Sweden has built in incentives promoting mothers' and fathers' equality in the upbringing of their children [70]. The involvement of fathers in parental training programs has many benefits [71] and may serve as a factor to increase resilience when encountering difficulties [72]. This fact only indicates the importance of actively involving both caregivers in the assessment and treatment of children with PP.

Very few young children were referred to the SPPC, but we chose to report statistics from them (*N* = 26), since research on younger children with PP is scarce and very much needed [02]. Unfortunately, the low number of children < 10 years did not allow for statistical analyses regarding differences between older and younger children. The low referral rate may be caused, e.g., by lower pain prevalence, fewer life consequences, or ongoing medical evaluations. This study adds to the very few studies describing characteristics of very young children with PP in tertiary care and including parent characteristics.

Considering that the estimated number of children with PP with impact on QoL and functioning in the uptake area is over 20 000, very few children were referred to the only existing SPPC in southern Sweden during the period 2013–2021. Factors such as individual resilience, degree of impairment, limited access to proper care, and biases in the referral process might contribute to the small number of children referred. This needs to be further investigated in future studies.

In this study, only half of the children assessed were offered IPR, which appears problematic when there are so few SPPCs. About 25% were returned to the referring clinic either with alternative suggestions for treatment or because their pain and functional status had improved to the extent that they no longer required IPR. Although some children were returned to the referring clinic because they improved prior to their assessment, we argue that a biopsychosocial interdisciplinary assessment is still valuable in recognizing and validating the positive coping strategies and resilience factors demonstrated by the child and family. Other children are returned for treatment not because of issues primarily related to pain, but due to other concerns that necessitate different interventions, such as experiences of bullying or family‐related problems.

About 15% were referred to psychiatry for assessment. This finding indicates that many children experience significant psychological distress, which is not necessarily a contraindication for IPR. If depression or anxiety is severe enough to include suicidal ideation, engagement in self‐harming behaviors, or extreme low functional capacity leading to incapacity to leave home, primary intervention from child psychiatry is needed. However, these children can be reassessed for IPR at a later stage. ADHD or ASD are generally not barriers to IPR, although an evaluation may be necessary to ensure that appropriate interventions and support are provided, particularly in educational settings.

About 7% declined the offer of IPR. Expectations of other treatment were sometimes given as a reason, but others believe that the time and commitment required for a treatment program cannot be justified, given the long travel times and school absences. Rehabilitation in an alternative format, such as digital or hybrid treatment, may increase the demand for services from both families and referring clinicians.

The question about whether a child benefits most from IPR or from other specific treatments before IPR is offered warrants further discussion. In our view, the degree to which the child is impacted in terms of daily and emotional functioning is the critical question rather than whether other treatments have already been attempted. This approach helps to avoid delays that may risk further functional impairment. The results of this study suggest that the complexity of pain and its impact on function tends to increase with age, which may, if not addressed, carry a risk of continued problems into adulthood. Further research is needed to enhance our understanding of which children require which specific interventions to better tailor care across different levels of healthcare.

Even if the study benefits from the inclusion of more than 500 patients with a large selection of measures completed by both parents and children, the findings must still be viewed within the context of certain limitations. Our findings cannot be generalized to children with PP in other clinical contexts. Mainly mothers participated, which could affect our interpretation of results concerning parents. Parent by‐proxy reports of younger children are also necessary, but a problematic method that raises obvious concerns. Still, results are strengthened by the inclusion of both children and parents in the same study, both approached from a biopsychosocial perspective, and by the relatively large number of participants.

We used self‐report and symptoms recorded in the medical records to identify symptoms of psychiatric comorbidities. Hence, we cannot ensure that the participants had been clinically diagnosed with a psychiatric disorder. Even if the use of self‐report is practical and standard practice, it only allows for a provisional diagnosis. Future studies should employ structured diagnostic interviews to identify psychiatric comorbidities.

Our retrospective descriptive data design does not allow for causality analyses. There might be a risk of bias since the clinician involved in the primary assessment is the first author. The competences of the SPPC and the instruments used in the study agree with the new suggested core outcome set of measures for pediatric pain studies as far as possible [5, 6, 17]. Nevertheless, a few non‐validated questionnaires were used in the absence of validated instruments in Swedish, emphasizing the need to validate instruments in different languages and contexts.

Nociplastic pain has recently been introduced as a type of pain, and since its introduction, the use of idiopathic pain as a descriptor of a pain type is more sporadic, also in our database. Most of the children included in this study were assessed prior to the introduction of nociplastic pain. We deemed it not possible to make a fair judgment about whether the pain type was nociplastic or idiopathic retrospectively. Therefore, we decided to combine them, as both concepts refer to a pain type that differs from the nociceptive and the neuropathic pain types, for instance, in terms of preferred treatment, e.g., pharmacological treatment.

This study adds to the knowledge of problems faced by children with PP in a Swedish context, which can possibly help the development of future interventions and increase availability to help even outside tertiary care for those not offered rehabilitation today. Tailored interventions of rehabilitation from a biopsychosocial perspective are recommended to improve functioning, including education on pain, parental aspects, and addressing psychiatric comorbidities, with special focus on ADHD or ASD symptoms.

More thorough information to referring physicians about evaluations and information needed before referring to tertiary pain clinics could potentially help set the right expectations for further care and reduce the risk of diagnostic uncertainty.

## Conflicts of Interest

The authors declare no conflicts of interest.

## Data Availability

The data that support the findings of this study are available on request from the corresponding author. The data are not publicly available due to privacy or ethical restrictions.
